# *rac*-6-Hy­droxy-2,5,7,8-tetra­methyl­chroman-2-carboxamide from synchrotron data

**DOI:** 10.1107/S1600536811002807

**Published:** 2011-01-29

**Authors:** Krzysztof Brzezinski, Zbigniew Dauter, Aneta Baj, Piotr Wałejko, Stanisław Witkowski

**Affiliations:** a Synchrotron Radiation Research Section, MCL, National Cancer Institute, Argonne National Laboratory, Biosciences Division, Bldg 202, Argonne, IL 60439, USA; b Institute of Chemistry, University of Białystok, Piłsudskiego 11/4, 15-443 Białystok, Poland

## Abstract

The crystal structure of the title water-soluble analogue of vitamin E, trolox amide, C_14_H_19_NO_3_, solved and refined against synchrotron diffraction data, contains two mol­ecules in the asymmetric unit. In both molecules, the heterocyclic ring is in a half-chair conformation. The crystal packing features a herring-bone pattern generated by N—H⋯O hydrogen bonds between the hy­droxy and amide groups. O—H⋯O hydrogen bonds also occur.

## Related literature

For background to the chemistry of trolox, its substituted amides and their applications as anti­oxidants and anti-inflamatory agents, see: Ross *et al.* (1995 ▶); Scott *et al.* (1974 ▶); Cort *et al.* (1975 ▶); Cohen *et al.* (1981 ▶); Walther *et al.* (1991 ▶); Silver *et al.* (1992 ▶); Netscher & Gautschi (1992 ▶); Van Ginkel *et al.* (1992) ▶; Moulin *et al.* (1998 ▶); Vajragupta *et al.* (2000 ▶); Koufaki *et al.* (2010 ▶). For the use of trolox as an inter­mediate for the synthesis of natural tocols such as vitamin E and α-tocotrienol, see: Cohen *et al.* (1979 ▶); Hyatt & Skelton (1997 ▶); Sakito & Suzokamo (1982 ▶); Sugai *et al.* (1991 ▶).
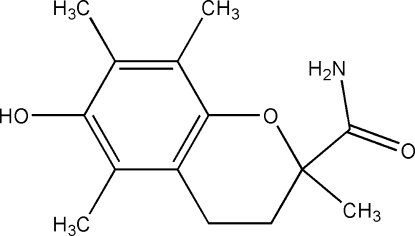


## Experimental

### 

#### Crystal data

C_14_H_19_NO_3_
*M* *_r_* = 249.31
Monoclinic, 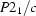

*a* = 9.11 (1) Å
*b* = 17.92 (2) Å
*c* = 15.95 (1) Å
β = 100.43 (1)°
*V* = 2561 (4) Å^3^
*Z* = 8
Synchrotron radiation
λ = 0.59040 Å
μ = 0.06 mm^−1^
*T* = 100 K
0.2 × 0.05 × 0.04 mm

#### Data collection

MAR315 CCD diffractometer
Absorption correction: multi-scan (*SCALEPACK*; Otwinowski & Minor, 2003 ▶) *T*_min_ = 0.988, *T*_max_ = 0.997
14016 measured reflections
6360 independent reflections
5153 reflections with *I* > 2σ(*I*)
*R*_int_ = 0.030

#### Refinement

*R*[*F* ^2^ > 2σ(*F* ^2^)] = 0.050
*wR*(*F* ^2^) = 0.147
*S* = 1.08
6360 reflections
327 parameters
H-atom parameters constrained
Δρ_max_ = 0.41 e Å^−3^
Δρ_min_ = −0.25 e Å^−3^

### 

Data collection: *NECAT APS beamline software*; cell refinement: *HKL-2000* (Otwinowski & Minor, 1997 ▶); data reduction: *HKL-2000*; program(s) used to solve structure: *SHELXD* (Sheldrick, 2008 ▶); program(s) used to refine structure: *SHELXL97* (Sheldrick, 2008 ▶); molecular graphics: *ORTEP-3* (Farrugia, 1997 ▶) and *pyMOL* (DeLano, 2002 ▶); software used to prepare material for publication: *SHELXL97*.

## Supplementary Material

Crystal structure: contains datablocks global, I. DOI: 10.1107/S1600536811002807/kp2304sup1.cif

Structure factors: contains datablocks I. DOI: 10.1107/S1600536811002807/kp2304Isup2.hkl

Additional supplementary materials:  crystallographic information; 3D view; checkCIF report

## Figures and Tables

**Table 1:** Hydrogen-bond geometry (Å, °)

*D*—H⋯*A*	*D*—H	H⋯*A*	*D*⋯*A*	*D*—H⋯*A*
N13—H13*A*⋯O16^i^	0.88	2.13	2.971 (2)	160
N13—H13*B*⋯O32^ii^	0.88	2.49	2.896 (3)	109
O16—H16*A*⋯O32^iii^	0.84	1.91	2.631 (2)	143
N33—H33*B*⋯O12	0.88	2.29	2.861 (3)	123
N33—H33*A*⋯O36^i^	0.88	2.53	3.281 (3)	143
O36—H36*A*⋯O12^iv^	0.84	1.91	2.727 (2)	165

Symmetry codes: (i) 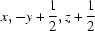; (ii) 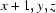; (iii) 
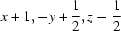
; (iv) 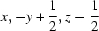.

**Fig. 1.**
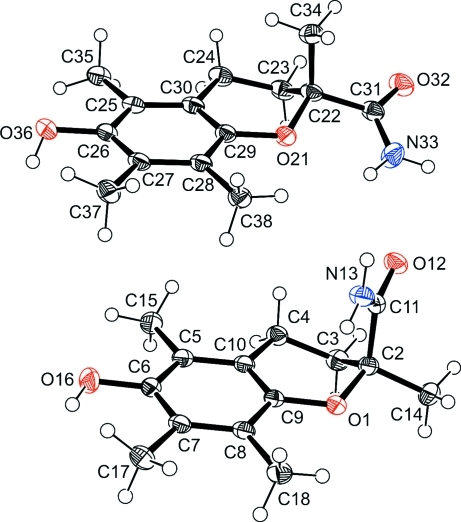
The view of the asymmetric unit of I. Displacement ellipsoids are drawn at the 50% probability level.

**Fig. 2.**
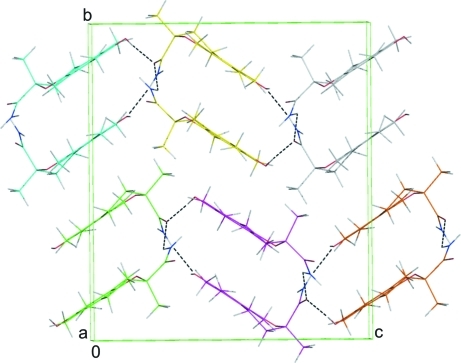
Crystal packing with hydrogen bonds (dashed lines) viewed along the a axis.

## supplementary crystallographic information

### Comment

Trolox is a water-soluble analog of α-tocopherol (vitamin E), in which lipophilic side chain was replaced with carboxylic group (Ross *et al.*, 1995). Owing to its high radical scavenging activity it is often used as a model compound for investigation of some aspects of vitamin E biological activity as well as for structural studies. It is commercially available in both enantiomerically pure forms, and is an important intermediate for the synthesis of natural tocols such as vitamin E and α-tocotrienol (Cohen *et al.*, 1979; Sakito *et al.*, 1982; Sugai *et al.*, 1991); Hyatt & Skelton, 1997).

The asymmetric unit contains two molecules differing in chirality of C2 (C22) atoms and in conformation of dihydropyranyl ring of chroman system. In both molecules the heterocyclic ring is in a half-chair conformation but the two out of plane atoms (C2 and C3 or C22 and C23) have the inverted configuration (Fig. 2). In one molecule the methyl group is axial and amide group is equatorial, whereas in the second molecule this arrangement is opposite. The average planes of the two unique molecules are highly parallel, but their aromatic rings are shifted and do not participate in effective π-stacking interactions. Together with their centrosymmetric mates they form columns of molecules extending along the *b* axis. The presence of the neighbouring columns related by the 2_1_ axes or c-glide planes results in the overall herring-bone type arrangement of molecules in the crystal (Fig. 2). Each molecule participates in three intermolecular hydrogen bonds engaging both amide oxygen and nitrogen atoms and the hydroxy group. The hydrogen bond network connects molecules of the adjacent columns (Table 1 and Fig. 2).

### Experimental

The title compound was obtained from *RS*-trolox in two-step synthesis *via* acyl chloride (SOCl_2_, DMF) followed by aminolysis (NH_3_ in CHCl_3_). After purification by column-flush chromatography pure crystalline compound was obtained (80% yield); mp 491–493 K; 1H NMR (MeOH-d4): δ 2.65–2.56 (m, 2H), 2.34–2.28 (m, 1H), 2.17 (s, 6H), 2.09 (s, 3H), 1.88–1.81 (m, 1H), 1.50 (s, 3H) p.p.m.; 13 C NMR (MeOH-d4) 178.6, 145.7, 144.3, 123.4, 121.4, 120.8, 117.1, 72.6, 29.4, 23.3, 20.1, 11.3, 10.7, 10.4 p.p.m.; IR (KBr): 3493; 3372; 2927; 1647; 1578 cm_-1_; ESI – MS: 272 (*M*^+^Na^+^). The crystallization was carried out at room temperature by slow evaporation of acetone solution of 6-hydroxy-2,5,7,8- tetramethylchroman-2-carboxamide.

### Refinement

All hydrogen atoms were constrained to idealized positions with C—H distances fixed at 0.98–0.99 Å and N—H distances fixed at 0.88 Å and *U*_iso_(H) = 1.5*U*_eq_(C) for methyl and hydroxy hydrogen atoms and 1.2*U*_eq_(C) for others.

### Crystal data

**Table:**

C_14_H_19_NO_3_	*F*(000) = 1072
*M**_r_* = 249.31	*D*_x_ = 1.293 Mg m^−^^3^
Monoclinic, *P*2_1_/*c*	Melting point: 492 K
Hall symbol: -P 2ybc	Synchrotron radiation, λ = 0.59040 Å
*a* = 9.11 (1) Å	Cell parameters from 6360 reflections
*b* = 17.92 (2) Å	θ = 1.4–23.5°
*c* = 15.95 (1) Å	µ = 0.06 mm^−^^1^
β = 100.43 (1)°	*T* = 100 K
*V* = 2561 (4) Å^3^	Needle, colourless
*Z* = 8	0.2 × 0.05 × 0.04 mm

### Data collection

**Table:**

MAR315 CCD diffractometer	6360 independent reflections
Radiation source: NECAT 24ID-C synchrotron beamline APS, USA	5153 reflections with *I* > 2σ(*I*)
Si111 double crystal	*R*_int_ = 0.030
ω scans	θ_max_ = 23.5°, θ_min_ = 1.4°
Absorption correction: multi-scan (*SCALEPACK*; Otwinowski *et al.*, 2003)	*h* = 0→12
*T*_min_ = 0.988, *T*_max_ = 0.997	*k* = 0→24
14016 measured reflections	*l* = −21→20

### Refinement

**Table:**

Refinement on *F*^2^	Primary atom site location: structure-invariant direct methods
Least-squares matrix: full	Secondary atom site location: difference Fourier map
*R*[*F*^2^ > 2σ(*F*^2^)] = 0.050	Hydrogen site location: inferred from neighbouring sites
*wR*(*F*^2^) = 0.147	H-atom parameters constrained
*S* = 1.08	*w* = 1/[σ^2^(*F*_o_^2^) + (0.0846*P*)^2^ + 0.4358*P*] where *P* = (*F*_o_^2^ + 2*F*_c_^2^)/3
6360 reflections	(Δ/σ)_max_ < 0.001
327 parameters	Δρ_max_ = 0.41 e Å^−^^3^
0 restraints	Δρ_min_ = −0.25 e Å^−^^3^

### Special details

**Table:**

Experimental. The crystal was mounted with vaseline on a pin attached capillary. Upon mounting, the crystal was quenched to 100 K in a nitrogen-gas stream supplied by an Oxford Cryo-Jet. Diffraction data were measured at the station 24-ID—C of the APS synchrotron by rotation method.
Geometry. All e.s.d.'s are estimated using the full covariance matrix. The cell e.s.d.'s are taken into account individually in the estimation of e.s.d.'s in distances, angles and torsion angles; correlations between e.s.d.'s in cell parameters are only used when they are defined by crystal symmetry. An approximate (isotropic) treatment of cell e.s.d.'s is used for estimating e.s.d.'s involving l.s. planes.
Refinement. Refinement of *F*^2^ against all reflections. The weighted *R*-factor *wR* and goodness of fit *S* are based on *F*^2^, conventional *R*-factors *R* are based on *F*, with *F* set to zero for negative *F*^2^. The threshold expression of *F*^2^ > 2σ(*F*^2^) is used only for calculating *R*-factors *etc*. and is not relevant to the choice of reflections for refinement. *R*-factors based on *F*^2^ are statistically about twice as large as those based on *F*.

### Fractional atomic coordinates and isotropic or equivalent isotropic displacement parameters (Å^2^)

**Table:**

	*x*	*y*	*z*	*U*_iso_*/*U*_eq_	
O1	0.61019 (10)	0.45827 (5)	0.16067 (5)	0.0245 (2)	
C2	0.47454 (14)	0.45990 (7)	0.19461 (8)	0.0237 (3)	
C3	0.34104 (15)	0.47162 (7)	0.12365 (8)	0.0268 (3)	
H3A	0.3515	0.5198	0.0950	0.032*	
H3B	0.2487	0.4736	0.1481	0.032*	
C4	0.32903 (14)	0.40842 (7)	0.05855 (8)	0.0247 (3)	
H4A	0.2887	0.3633	0.0822	0.030*	
H4B	0.2587	0.4231	0.0063	0.030*	
C5	0.48962 (14)	0.34738 (7)	−0.03638 (8)	0.0231 (3)	
C6	0.62994 (14)	0.33351 (7)	−0.05616 (8)	0.0235 (3)	
C7	0.76145 (14)	0.36122 (7)	−0.00604 (8)	0.0232 (3)	
C8	0.75096 (14)	0.40469 (7)	0.06572 (8)	0.0224 (2)	
C9	0.61017 (14)	0.41713 (6)	0.08636 (7)	0.0215 (2)	
C10	0.47925 (14)	0.39050 (7)	0.03598 (8)	0.0217 (2)	
C11	0.45591 (14)	0.38690 (7)	0.24307 (8)	0.0233 (3)	
O12	0.33708 (11)	0.37440 (5)	0.26791 (6)	0.0303 (2)	
N13	0.57098 (13)	0.34076 (6)	0.25872 (7)	0.0271 (2)	
H13A	0.5647	0.2995	0.2877	0.033*	
H13B	0.6538	0.3514	0.2401	0.033*	
C14	0.49388 (17)	0.52489 (7)	0.25803 (9)	0.0312 (3)	
H14A	0.4095	0.5259	0.2884	0.047*	
H14B	0.4975	0.5720	0.2272	0.047*	
H14C	0.5870	0.5184	0.2991	0.047*	
C15	0.35081 (15)	0.31770 (8)	−0.09261 (9)	0.0299 (3)	
H15A	0.3000	0.3584	−0.1274	0.045*	
H15B	0.2838	0.2968	−0.0570	0.045*	
H15C	0.3784	0.2787	−0.1299	0.045*	
O16	0.63187 (11)	0.29210 (6)	−0.12892 (6)	0.0325 (2)	
H16A	0.7183	0.2925	−0.1404	0.049*	
C17	0.91159 (15)	0.34490 (8)	−0.02946 (9)	0.0304 (3)	
H17A	0.9123	0.2938	−0.0513	0.046*	
H17B	0.9898	0.3502	0.0212	0.046*	
H17C	0.9301	0.3801	−0.0734	0.046*	
C18	0.88847 (15)	0.43836 (8)	0.12025 (9)	0.0302 (3)	
H18A	0.8581	0.4729	0.1615	0.045*	
H18B	0.9462	0.4654	0.0838	0.045*	
H18C	0.9500	0.3985	0.1507	0.045*	
O21	0.09463 (10)	0.20376 (5)	0.15518 (6)	0.0261 (2)	
C22	−0.03768 (14)	0.18816 (7)	0.18944 (8)	0.0246 (3)	
C23	−0.17485 (14)	0.19491 (8)	0.11915 (9)	0.0277 (3)	
H23A	−0.1817	0.2464	0.0963	0.033*	
H23B	−0.2658	0.1849	0.1431	0.033*	
C24	−0.16637 (14)	0.13979 (8)	0.04698 (9)	0.0278 (3)	
H24A	−0.2001	0.0901	0.0629	0.033*	
H24B	−0.2350	0.1563	−0.0051	0.033*	
C25	0.01411 (14)	0.09450 (7)	−0.04523 (8)	0.0242 (3)	
C26	0.16025 (14)	0.08797 (7)	−0.06026 (8)	0.0244 (3)	
C27	0.28233 (14)	0.11907 (7)	−0.00506 (8)	0.0236 (3)	
C28	0.25737 (14)	0.15918 (7)	0.06677 (8)	0.0229 (3)	
C29	0.11127 (14)	0.16416 (7)	0.08231 (8)	0.0228 (3)	
C30	−0.01047 (14)	0.13307 (7)	0.02768 (8)	0.0235 (3)	
C31	−0.04526 (15)	0.24820 (7)	0.25651 (8)	0.0270 (3)	
O32	−0.15782 (11)	0.25283 (6)	0.28939 (6)	0.0353 (2)	
N33	0.07066 (14)	0.29294 (7)	0.27773 (8)	0.0360 (3)	
H33A	0.0696	0.3277	0.3166	0.043*	
H33B	0.1490	0.2880	0.2530	0.043*	
C34	−0.02493 (16)	0.11133 (7)	0.23290 (9)	0.0300 (3)	
H34A	−0.1073	0.1049	0.2642	0.045*	
H34B	0.0704	0.1079	0.2727	0.045*	
H34C	−0.0298	0.0722	0.1896	0.045*	
C35	−0.11451 (15)	0.06001 (8)	−0.10581 (8)	0.0291 (3)	
H35A	−0.0827	0.0480	−0.1597	0.044*	
H35B	−0.1979	0.0954	−0.1164	0.044*	
H35C	−0.1465	0.0143	−0.0806	0.044*	
O36	0.17961 (12)	0.04723 (6)	−0.13154 (6)	0.0315 (2)	
H36A	0.2416	0.0691	−0.1561	0.047*	
C37	0.43909 (15)	0.10894 (8)	−0.02105 (9)	0.0294 (3)	
H37A	0.4698	0.1537	−0.0486	0.044*	
H37B	0.4426	0.0657	−0.0582	0.044*	
H37C	0.5069	0.1008	0.0333	0.044*	
C38	0.38410 (14)	0.19683 (7)	0.12563 (8)	0.0268 (3)	
H38A	0.4358	0.2309	0.0927	0.040*	
H38B	0.4543	0.1590	0.1532	0.040*	
H38C	0.3445	0.2250	0.1692	0.040*	

### Atomic displacement parameters (Å^2^)

**Table:**

	*U*^11^	*U*^22^	*U*^33^	*U*^12^	*U*^13^	*U*^23^
O1	0.0261 (5)	0.0241 (4)	0.0258 (4)	−0.0053 (3)	0.0114 (4)	−0.0031 (3)
C2	0.0277 (6)	0.0182 (5)	0.0282 (6)	0.0000 (5)	0.0130 (5)	−0.0012 (4)
C3	0.0281 (6)	0.0216 (6)	0.0326 (7)	0.0044 (5)	0.0104 (5)	0.0027 (5)
C4	0.0230 (6)	0.0242 (6)	0.0285 (6)	−0.0001 (5)	0.0091 (5)	0.0002 (5)
C5	0.0243 (6)	0.0203 (6)	0.0262 (6)	−0.0031 (5)	0.0083 (5)	0.0012 (5)
C6	0.0266 (6)	0.0203 (6)	0.0260 (6)	−0.0020 (5)	0.0112 (5)	−0.0013 (4)
C7	0.0231 (6)	0.0214 (6)	0.0273 (6)	−0.0016 (5)	0.0104 (5)	0.0033 (5)
C8	0.0231 (6)	0.0203 (6)	0.0255 (6)	−0.0023 (5)	0.0086 (5)	0.0040 (4)
C9	0.0256 (6)	0.0178 (5)	0.0232 (6)	−0.0030 (4)	0.0099 (5)	0.0010 (4)
C10	0.0225 (6)	0.0185 (5)	0.0261 (6)	−0.0015 (4)	0.0097 (5)	0.0025 (4)
C11	0.0280 (6)	0.0214 (6)	0.0225 (6)	−0.0009 (5)	0.0103 (5)	−0.0023 (4)
O12	0.0315 (5)	0.0293 (5)	0.0349 (5)	−0.0014 (4)	0.0189 (4)	0.0001 (4)
N13	0.0286 (6)	0.0229 (5)	0.0320 (6)	0.0012 (4)	0.0117 (5)	0.0041 (4)
C14	0.0400 (8)	0.0214 (6)	0.0349 (7)	−0.0012 (5)	0.0140 (6)	−0.0062 (5)
C15	0.0259 (7)	0.0327 (7)	0.0323 (7)	−0.0054 (5)	0.0087 (5)	−0.0049 (5)
O16	0.0290 (5)	0.0368 (5)	0.0349 (5)	−0.0045 (4)	0.0144 (4)	−0.0126 (4)
C17	0.0240 (6)	0.0363 (7)	0.0340 (7)	−0.0015 (5)	0.0136 (5)	−0.0005 (6)
C18	0.0252 (7)	0.0353 (7)	0.0309 (7)	−0.0073 (5)	0.0073 (5)	−0.0018 (5)
O21	0.0220 (4)	0.0269 (5)	0.0332 (5)	−0.0036 (3)	0.0152 (4)	−0.0040 (4)
C22	0.0223 (6)	0.0229 (6)	0.0322 (6)	0.0006 (5)	0.0144 (5)	0.0041 (5)
C23	0.0209 (6)	0.0299 (7)	0.0353 (7)	0.0029 (5)	0.0131 (5)	0.0063 (5)
C24	0.0193 (6)	0.0312 (7)	0.0342 (7)	−0.0012 (5)	0.0086 (5)	0.0048 (5)
C25	0.0245 (6)	0.0224 (6)	0.0269 (6)	−0.0027 (5)	0.0078 (5)	0.0066 (5)
C26	0.0284 (6)	0.0222 (6)	0.0250 (6)	−0.0023 (5)	0.0113 (5)	0.0034 (5)
C27	0.0226 (6)	0.0225 (6)	0.0285 (6)	−0.0011 (5)	0.0120 (5)	0.0043 (5)
C28	0.0214 (6)	0.0208 (6)	0.0286 (6)	−0.0011 (4)	0.0102 (5)	0.0036 (5)
C29	0.0226 (6)	0.0203 (6)	0.0279 (6)	0.0001 (4)	0.0112 (5)	0.0023 (4)
C30	0.0200 (6)	0.0226 (6)	0.0298 (6)	−0.0006 (4)	0.0098 (5)	0.0056 (5)
C31	0.0278 (7)	0.0237 (6)	0.0330 (7)	0.0047 (5)	0.0146 (5)	0.0040 (5)
O32	0.0342 (6)	0.0344 (5)	0.0434 (6)	0.0027 (4)	0.0238 (5)	0.0012 (4)
N33	0.0321 (6)	0.0329 (6)	0.0473 (7)	−0.0029 (5)	0.0189 (5)	−0.0144 (5)
C34	0.0332 (7)	0.0234 (6)	0.0342 (7)	−0.0021 (5)	0.0082 (6)	0.0041 (5)
C35	0.0282 (7)	0.0302 (7)	0.0292 (6)	−0.0047 (5)	0.0056 (5)	0.0049 (5)
O36	0.0366 (6)	0.0325 (5)	0.0296 (5)	−0.0084 (4)	0.0173 (4)	−0.0026 (4)
C37	0.0246 (6)	0.0331 (7)	0.0339 (7)	−0.0005 (5)	0.0143 (5)	−0.0004 (5)
C38	0.0224 (6)	0.0274 (6)	0.0326 (7)	−0.0026 (5)	0.0104 (5)	−0.0008 (5)

### Geometric parameters (Å, °)

**Table:**

O1—C9	1.3958 (18)	O21—C29	1.3933 (18)
O1—C2	1.4364 (19)	O21—C22	1.4381 (18)
C2—C3	1.518 (2)	C22—C23	1.525 (2)
C2—C14	1.532 (2)	C22—C31	1.527 (2)
C2—C11	1.544 (2)	C22—C34	1.536 (2)
C3—C4	1.527 (2)	C23—C24	1.529 (2)
C3—H3A	0.9900	C23—H23A	0.9900
C3—H3B	0.9900	C23—H23B	0.9900
C4—C10	1.511 (2)	C24—C30	1.511 (2)
C4—H4A	0.9900	C24—H24A	0.9900
C4—H4B	0.9900	C24—H24B	0.9900
C5—C6	1.393 (2)	C25—C26	1.400 (2)
C5—C10	1.406 (2)	C25—C30	1.405 (2)
C5—C15	1.509 (2)	C25—C35	1.510 (2)
C6—O16	1.3803 (18)	C26—O36	1.3889 (18)
C6—C7	1.405 (2)	C26—C27	1.403 (2)
C7—C8	1.402 (2)	C27—C28	1.406 (2)
C7—C17	1.510 (2)	C27—C37	1.506 (2)
C8—C9	1.399 (2)	C28—C29	1.400 (2)
C8—C18	1.515 (2)	C28—C38	1.509 (2)
C9—C10	1.396 (2)	C29—C30	1.396 (2)
C11—O12	1.2387 (18)	C31—O32	1.2362 (18)
C11—N13	1.3230 (19)	C31—N33	1.320 (2)
N13—H13A	0.8800	N33—H33A	0.8800
N13—H13B	0.8800	N33—H33B	0.8800
C14—H14A	0.9800	C34—H34A	0.9800
C14—H14B	0.9800	C34—H34B	0.9800
C14—H14C	0.9800	C34—H34C	0.9800
C15—H15A	0.9800	C35—H35A	0.9800
C15—H15B	0.9800	C35—H35B	0.9800
C15—H15C	0.9800	C35—H35C	0.9800
O16—H16A	0.8400	O36—H36A	0.8400
C17—H17A	0.9800	C37—H37A	0.9800
C17—H17B	0.9800	C37—H37B	0.9800
C17—H17C	0.9800	C37—H37C	0.9800
C18—H18A	0.9800	C38—H38A	0.9800
C18—H18B	0.9800	C38—H38B	0.9800
C18—H18C	0.9800	C38—H38C	0.9800
			
C9—O1—C2	117.57 (9)	C29—O21—C22	116.31 (10)
O1—C2—C3	110.46 (12)	O21—C22—C23	109.68 (12)
O1—C2—C14	105.09 (11)	O21—C22—C31	106.05 (10)
C3—C2—C14	111.59 (11)	C23—C22—C31	108.91 (11)
O1—C2—C11	110.57 (10)	O21—C22—C34	110.22 (10)
C3—C2—C11	110.15 (11)	C23—C22—C34	112.81 (11)
C14—C2—C11	108.87 (12)	C31—C22—C34	108.94 (12)
C2—C3—C4	110.69 (11)	C22—C23—C24	110.84 (11)
C2—C3—H3A	109.5	C22—C23—H23A	109.5
C4—C3—H3A	109.5	C24—C23—H23A	109.5
C2—C3—H3B	109.5	C22—C23—H23B	109.5
C4—C3—H3B	109.5	C24—C23—H23B	109.5
H3A—C3—H3B	108.1	H23A—C23—H23B	108.1
C10—C4—C3	111.29 (11)	C30—C24—C23	112.47 (11)
C10—C4—H4A	109.4	C30—C24—H24A	109.1
C3—C4—H4A	109.4	C23—C24—H24A	109.1
C10—C4—H4B	109.4	C30—C24—H24B	109.1
C3—C4—H4B	109.4	C23—C24—H24B	109.1
H4A—C4—H4B	108.0	H24A—C24—H24B	107.8
C6—C5—C10	118.98 (11)	C26—C25—C30	118.81 (11)
C6—C5—C15	120.47 (12)	C26—C25—C35	120.54 (13)
C10—C5—C15	120.54 (12)	C30—C25—C35	120.64 (12)
O16—C6—C5	115.99 (11)	O36—C26—C25	116.73 (11)
O16—C6—C7	121.94 (12)	O36—C26—C27	121.25 (12)
C5—C6—C7	122.06 (13)	C25—C26—C27	121.99 (13)
C8—C7—C6	118.93 (12)	C28—C27—C26	119.20 (12)
C8—C7—C17	120.54 (11)	C28—C27—C37	119.85 (11)
C6—C7—C17	120.53 (13)	C26—C27—C37	120.93 (13)
C9—C8—C7	118.79 (11)	C29—C28—C27	118.43 (11)
C9—C8—C18	119.98 (12)	C29—C28—C38	120.41 (12)
C7—C8—C18	121.23 (12)	C27—C28—C38	121.16 (12)
O1—C9—C8	115.15 (11)	O21—C29—C30	121.98 (12)
O1—C9—C10	122.55 (11)	O21—C29—C28	115.50 (11)
C8—C9—C10	122.30 (12)	C30—C29—C28	122.50 (13)
C9—C10—C5	118.88 (12)	C29—C30—C25	119.02 (12)
C9—C10—C4	120.42 (12)	C29—C30—C24	120.70 (12)
C5—C10—C4	120.70 (11)	C25—C30—C24	120.28 (11)
O12—C11—N13	122.36 (13)	O32—C31—N33	122.47 (14)
O12—C11—C2	119.66 (11)	O32—C31—C22	119.38 (12)
N13—C11—C2	117.95 (12)	N33—C31—C22	118.15 (12)
C11—N13—H13A	120.0	C31—N33—H33A	120.0
C11—N13—H13B	120.0	C31—N33—H33B	120.0
H13A—N13—H13B	120.0	H33A—N33—H33B	120.0
C2—C14—H14A	109.5	C22—C34—H34A	109.5
C2—C14—H14B	109.5	C22—C34—H34B	109.5
H14A—C14—H14B	109.5	H34A—C34—H34B	109.5
C2—C14—H14C	109.5	C22—C34—H34C	109.5
H14A—C14—H14C	109.5	H34A—C34—H34C	109.5
H14B—C14—H14C	109.5	H34B—C34—H34C	109.5
C5—C15—H15A	109.5	C25—C35—H35A	109.5
C5—C15—H15B	109.5	C25—C35—H35B	109.5
H15A—C15—H15B	109.5	H35A—C35—H35B	109.5
C5—C15—H15C	109.5	C25—C35—H35C	109.5
H15A—C15—H15C	109.5	H35A—C35—H35C	109.5
H15B—C15—H15C	109.5	H35B—C35—H35C	109.5
C6—O16—H16A	109.5	C26—O36—H36A	109.5
C7—C17—H17A	109.5	C27—C37—H37A	109.5
C7—C17—H17B	109.5	C27—C37—H37B	109.5
H17A—C17—H17B	109.5	H37A—C37—H37B	109.5
C7—C17—H17C	109.5	C27—C37—H37C	109.5
H17A—C17—H17C	109.5	H37A—C37—H37C	109.5
H17B—C17—H17C	109.5	H37B—C37—H37C	109.5
C8—C18—H18A	109.5	C28—C38—H38A	109.5
C8—C18—H18B	109.5	C28—C38—H38B	109.5
H18A—C18—H18B	109.5	H38A—C38—H38B	109.5
C8—C18—H18C	109.5	C28—C38—H38C	109.5
H18A—C18—H18C	109.5	H38A—C38—H38C	109.5
H18B—C18—H18C	109.5	H38B—C38—H38C	109.5
			
C9—O1—C2—C3	−43.88 (14)	C29—O21—C22—C23	−51.64 (14)
C9—O1—C2—C14	−164.37 (10)	C29—O21—C22—C31	−169.10 (10)
C9—O1—C2—C11	78.31 (14)	C29—O21—C22—C34	73.14 (14)
O1—C2—C3—C4	60.30 (13)	O21—C22—C23—C24	59.96 (14)
C14—C2—C3—C4	176.82 (11)	C31—C22—C23—C24	175.62 (10)
C11—C2—C3—C4	−62.14 (14)	C34—C22—C23—C24	−63.30 (15)
C2—C3—C4—C10	−44.86 (15)	C22—C23—C24—C30	−39.37 (15)
C10—C5—C6—O16	−178.75 (10)	C30—C25—C26—O36	177.90 (11)
C15—C5—C6—O16	0.21 (17)	C35—C25—C26—O36	−1.46 (17)
C10—C5—C6—C7	0.03 (18)	C30—C25—C26—C27	−0.36 (18)
C15—C5—C6—C7	178.99 (11)	C35—C25—C26—C27	−179.72 (11)
O16—C6—C7—C8	178.19 (11)	O36—C26—C27—C28	−179.33 (11)
C5—C6—C7—C8	−0.53 (18)	C25—C26—C27—C28	−1.15 (18)
O16—C6—C7—C17	−1.37 (18)	O36—C26—C27—C37	−0.33 (18)
C5—C6—C7—C17	179.92 (12)	C25—C26—C27—C37	177.85 (12)
C6—C7—C8—C9	1.86 (17)	C26—C27—C28—C29	2.41 (17)
C17—C7—C8—C9	−178.58 (11)	C37—C27—C28—C29	−176.60 (11)
C6—C7—C8—C18	−177.76 (11)	C26—C27—C28—C38	−176.99 (11)
C17—C7—C8—C18	1.80 (18)	C37—C27—C28—C38	4.00 (18)
C2—O1—C9—C8	−167.53 (10)	C22—O21—C29—C30	22.81 (16)
C2—O1—C9—C10	12.70 (16)	C22—O21—C29—C28	−158.56 (11)
C7—C8—C9—O1	177.40 (10)	C27—C28—C29—O21	179.10 (10)
C18—C8—C9—O1	−2.97 (16)	C38—C28—C29—O21	−1.49 (17)
C7—C8—C9—C10	−2.83 (18)	C27—C28—C29—C30	−2.28 (18)
C18—C8—C9—C10	176.80 (11)	C38—C28—C29—C30	177.13 (11)
O1—C9—C10—C5	−177.91 (10)	O21—C29—C30—C25	179.31 (11)
C8—C9—C10—C5	2.34 (18)	C28—C29—C30—C25	0.78 (18)
O1—C9—C10—C4	2.66 (17)	O21—C29—C30—C24	−1.47 (18)
C8—C9—C10—C4	−177.10 (11)	C28—C29—C30—C24	179.99 (11)
C6—C5—C10—C9	−0.90 (17)	C26—C25—C30—C29	0.56 (18)
C15—C5—C10—C9	−179.86 (11)	C35—C25—C30—C29	179.91 (11)
C6—C5—C10—C4	178.53 (11)	C26—C25—C30—C24	−178.66 (11)
C15—C5—C10—C4	−0.43 (18)	C35—C25—C30—C24	0.69 (18)
C3—C4—C10—C9	14.64 (16)	C23—C24—C30—C29	11.06 (17)
C3—C4—C10—C5	−164.78 (11)	C23—C24—C30—C25	−169.73 (11)
O1—C2—C11—O12	−171.35 (11)	O21—C22—C31—O32	171.96 (11)
C3—C2—C11—O12	−48.97 (16)	C23—C22—C31—O32	53.99 (16)
C14—C2—C11—O12	73.68 (15)	C34—C22—C31—O32	−69.42 (15)
O1—C2—C11—N13	10.66 (15)	O21—C22—C31—N33	−8.72 (16)
C3—C2—C11—N13	133.04 (13)	C23—C22—C31—N33	−126.69 (14)
C14—C2—C11—N13	−104.31 (13)	C34—C22—C31—N33	109.89 (14)

### Hydrogen-bond geometry (Å, °)

**Table:**

*D*—H···*A*	*D*—H	H···*A*	*D*···*A*	*D*—H···*A*
N13—H13A···O16^i^	0.88	2.13	2.971 (2)	160
N13—H13B···O32^ii^	0.88	2.49	2.896 (3)	109
O16—H16A···O32^iii^	0.84	1.91	2.631 (2)	143
N33—H33B···O12	0.88	2.29	2.861 (3)	123
N33—H33A···O36^i^	0.88	2.53	3.281 (3)	143
O36—H36A···O12^iv^	0.84	1.91	2.727 (2)	165

Symmetry codes: (i) *x*, −*y*+1/2, *z*+1/2; (ii) *x*+1, *y*, *z*; (iii) *x*+1, −*y*+1/2, *z*−1/2;  (iv) *x*, −*y*+1/2, *z*−1/2.
